# Supplementary material for: A gene drive does not spread easily in populations of the honey bee parasite Varroa destructor
Source: Apidologie. 2021 Oct 15;52(6):1112–27. doi: 10.1007/s13592-021-00891-5 (PMC8755698; doi:10.1007/s13592-021-00891-5)
Supplement: Supplementary file 1 — Supplementary file1 (PDF 1.44 MB) [file 13592_2021_891_MOESM1_ESM.pdf]

Supplementary Material

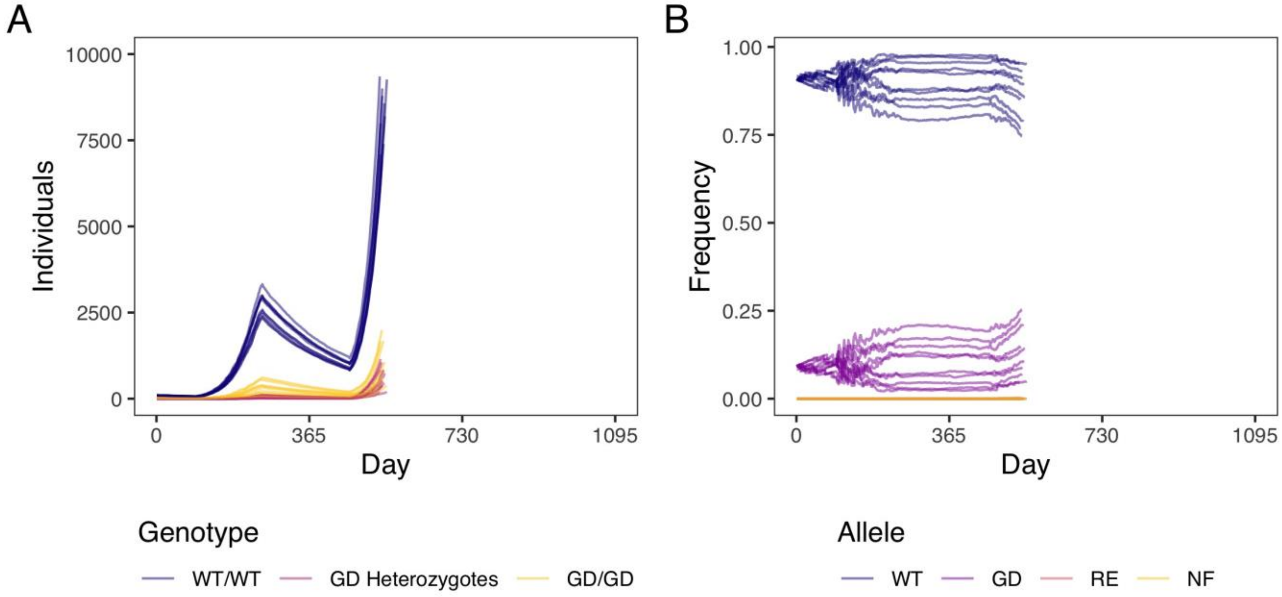

Figure S1. Model of varroa and gene drive spread as in Figure 2C and D, but with a 10 times larger starting population: 100 wild-type varroa instead of 10, and 10 gene drive varroa instead of 1. The initial population size is 100 wild-type varroa with 10 added homozygous gene drive varroa, giving an initial gene drive frequency of 0.09. For every set of parameters, we run 10 repetitions and stop the model when the varroa population size is over 10,000. **A)** Numbers of individuals with different genotypes. WT = wild-type and GD = gene drive. **B)** Frequencies of gene drive alleles. WT = wild-type, GD = gene drive, RE = resistant, and NF = non-functional.

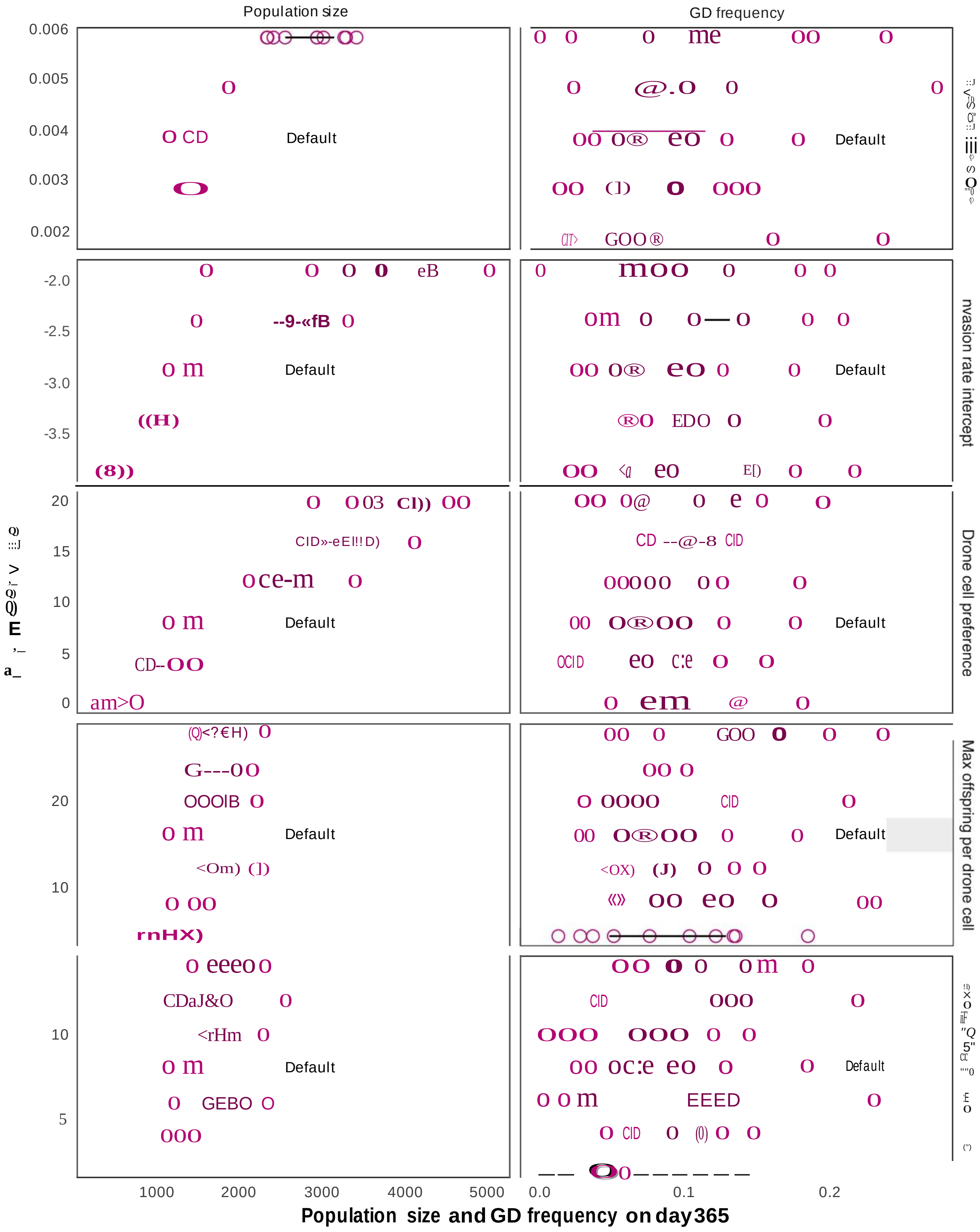

Figure 52. Sensitivity analysis of our varroa model shown in Figure 2A and D. We run the model for a year with a range of parameters and on day 365, we measure both population size and gene drive (GD) frequency to see which parameter has an influence. The initial population size is 100 wild-type varroa with 10 added homozygous gene drive varroa, giving an initial gene drive frequency of 0.09. We vary five parameters independently: invasion rate slope (see Equation 1), invasion rate intercept (see Equation 1), drone cell preference, max offspring per drone cell (see Equation 2), and max offspring per worker cell (see Equation 2). Pink circles indicate each repetition's outcome, the black lines represent the 95% confidence interval around the mean, and the grey bar and text "Default" indicate the default parameters that are supported by literature and are used in Figure 2 and all other figures. For every set of parameters, we run 10 repetitions and stop the model when the varroa population size is over 10,000.

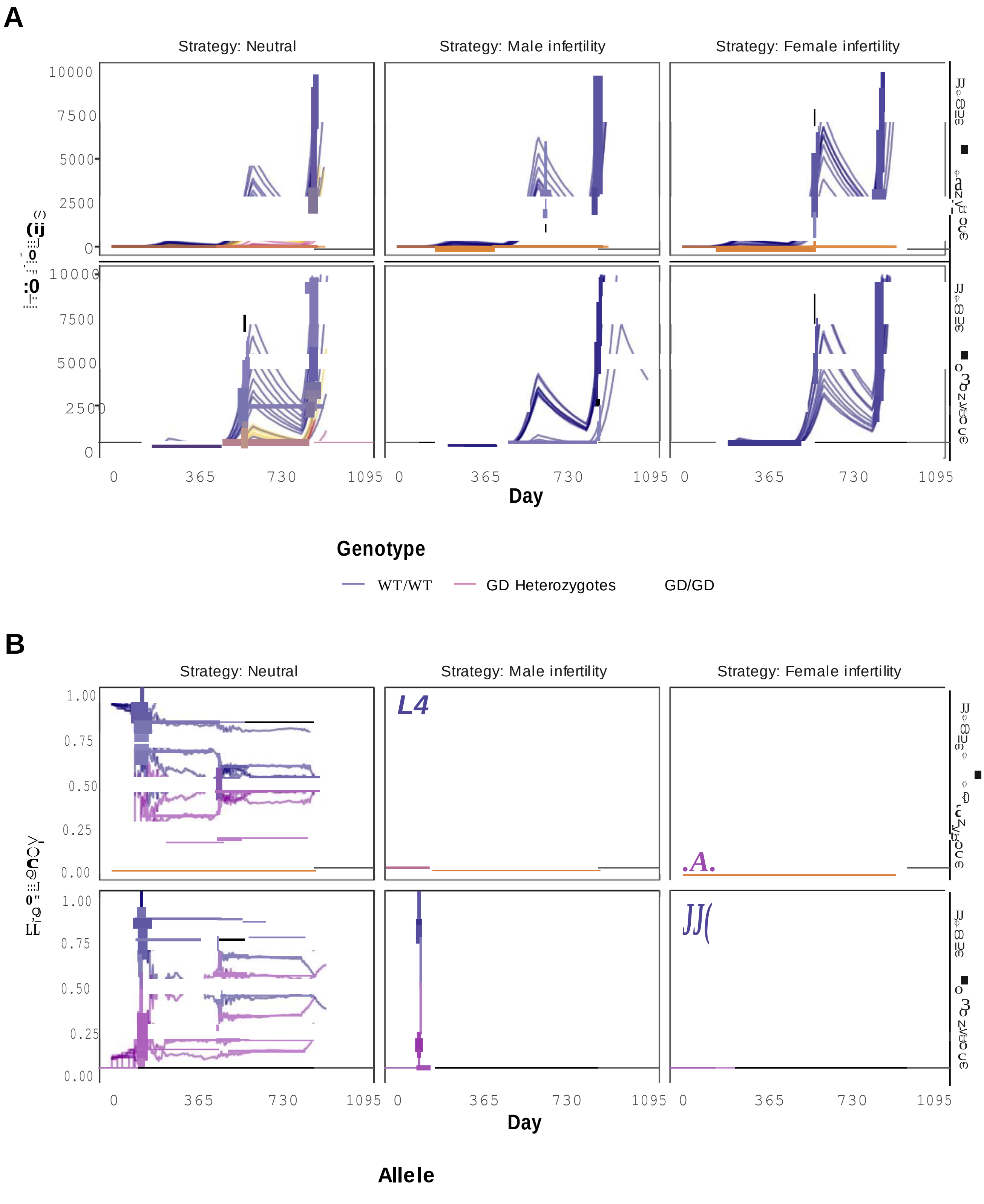

Figure 53. Model of varroa and gene drive spread as in Figure 2C and D, but besides a neutral gene drive, we also model a gene drive which, when homozygous or hemizygous, causes male or female infertility. Besides the release of homozygous females as in Figure 2C and D, we also model the release of heterozygous gene drive varroa females so the infertility does not immediately affect females. The initial population size is 10 wild-type varroa with 1 added gene drive varroa, giving an initial gene drive frequency of 0.09 for a homozygote release and 0.045 for a heterozygote release. For every set of parameters, we run 10 repetitions and stop the model when the varroa population size is over 10,000. **A)** Numbers of individuals with different genotypes. WT = wild-type and GD = gene drive. **B)** Frequencies of gene drive alleles. WT = wild-type, GD = gene drive, RE = resistant, and NF = non-functional.

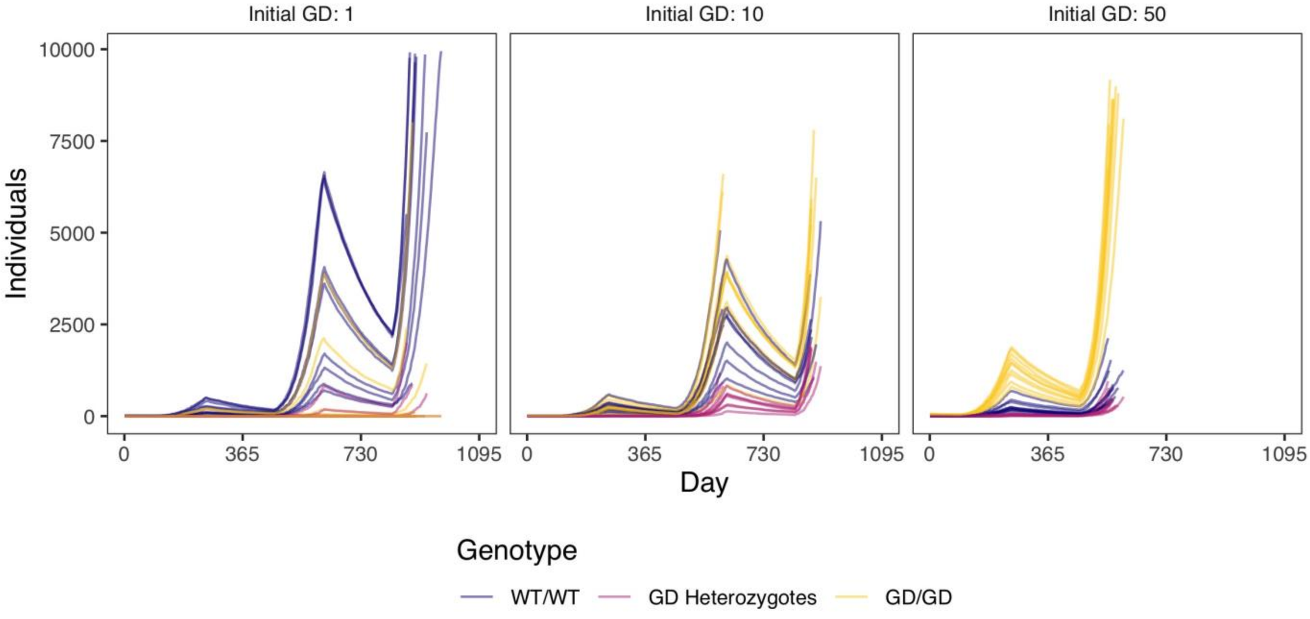

Figure S4. Numbers of individuals with three genotypes, corresponding to the allele frequencies in Figure 3 over three years with different gene drive introduction amounts. The initial population size is 10 wild-type varroa with 1, 10 or 50 added homozygous gene drive varroa, giving initial gene drive frequencies of 0.09, 0.50, and 0.83, respectively. WT = wild-type and GD = gene drive. For every set of parameters, we run 10 repetitions and stop the model when the varroa population size is over 10,000.

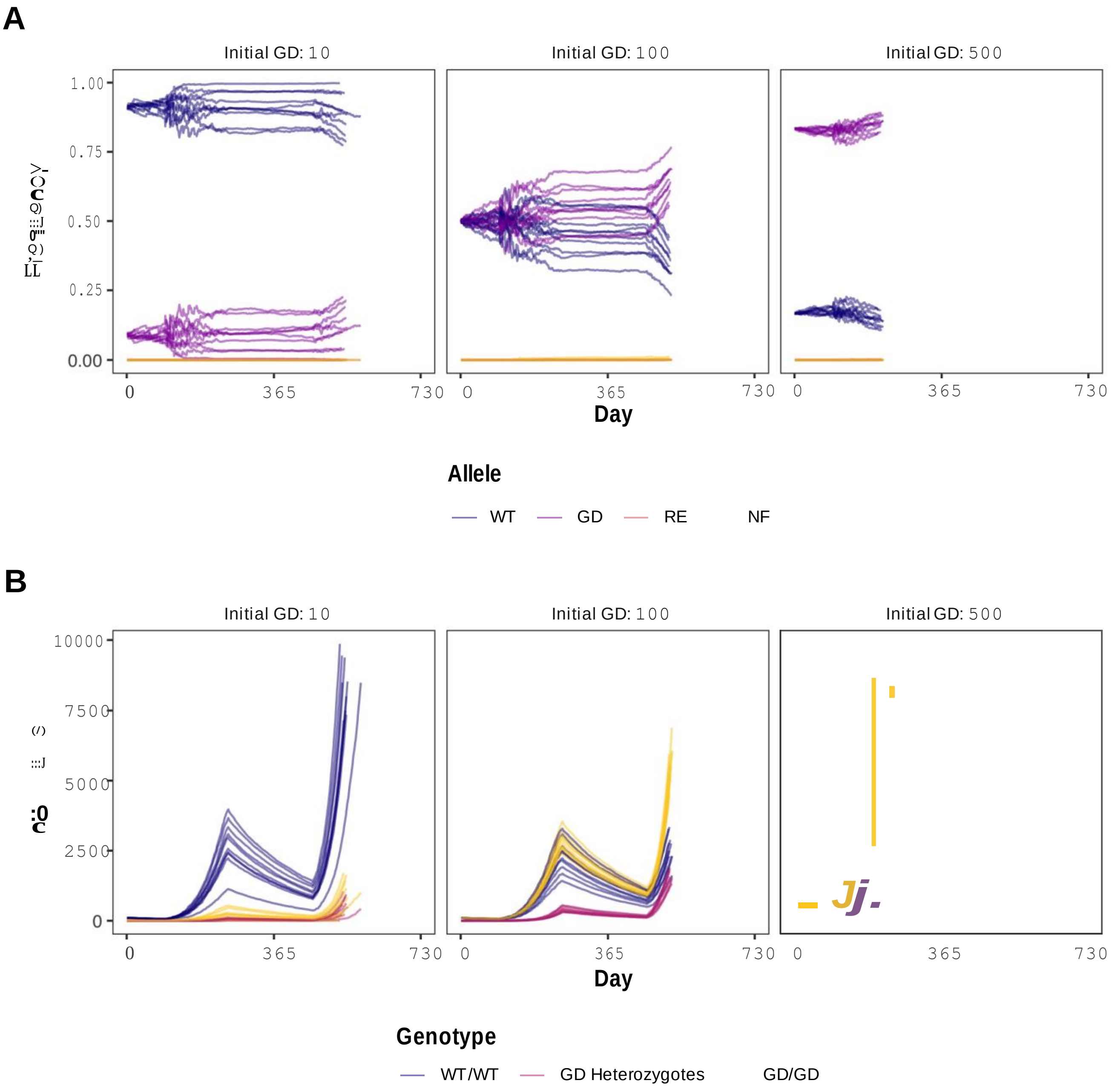

Figure 55. The same as Figure 3 and Figure 84, but with 10 times more initial varroa. The initial population size is 100 wild-type varroa with 10, 100 or 500 added homozygous gene drive varroa, respectively giving initial gene drive frequencies of 0.09, 0.50, and 0.83. For every set of parameters, we run 10 repetitions and stop the model when the varroa population size is over 10,000. **A)** Allele frequencies over three years with different gene drive introduction amounts. WT = wild-type, GD = gene drive, RE = resistant, and NF = non-functional. **B)** Numbers of individuals with three genotypes. WT = wild-type and GD = gene drive.

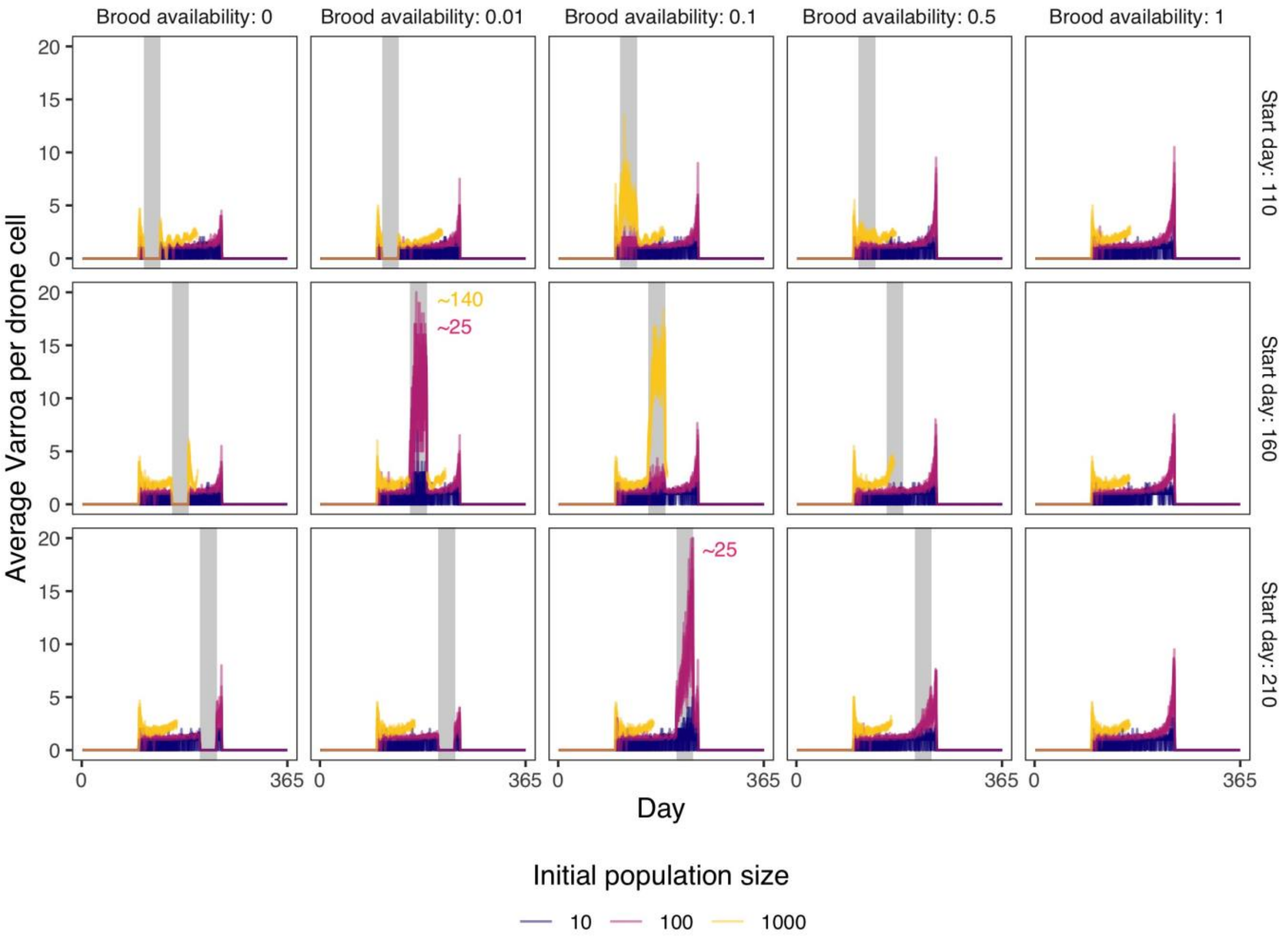

Figure S6. Average varroa per drone cell over time for different initial population sizes, given different amounts of brood cell availability (as a fraction of the normal amount) and different brood break starting days like in Figure 4. The grey bars indicate the brood break. The "~" in two plots indicates that values were higher than 20 and thus fall off the truncated y-axis to keep the plot interpretable. The number after the "~" roughly indicates the maximum of the truncated values. The initial population sizes were 10, 100, or 1000 wild-type varroa with the same number of gene drive varroa on top of that, giving initial gene drive frequencies of 0.5. For every set of parameters, we run 10 repetitions and stop the model when the varroa population size is over 10,000.

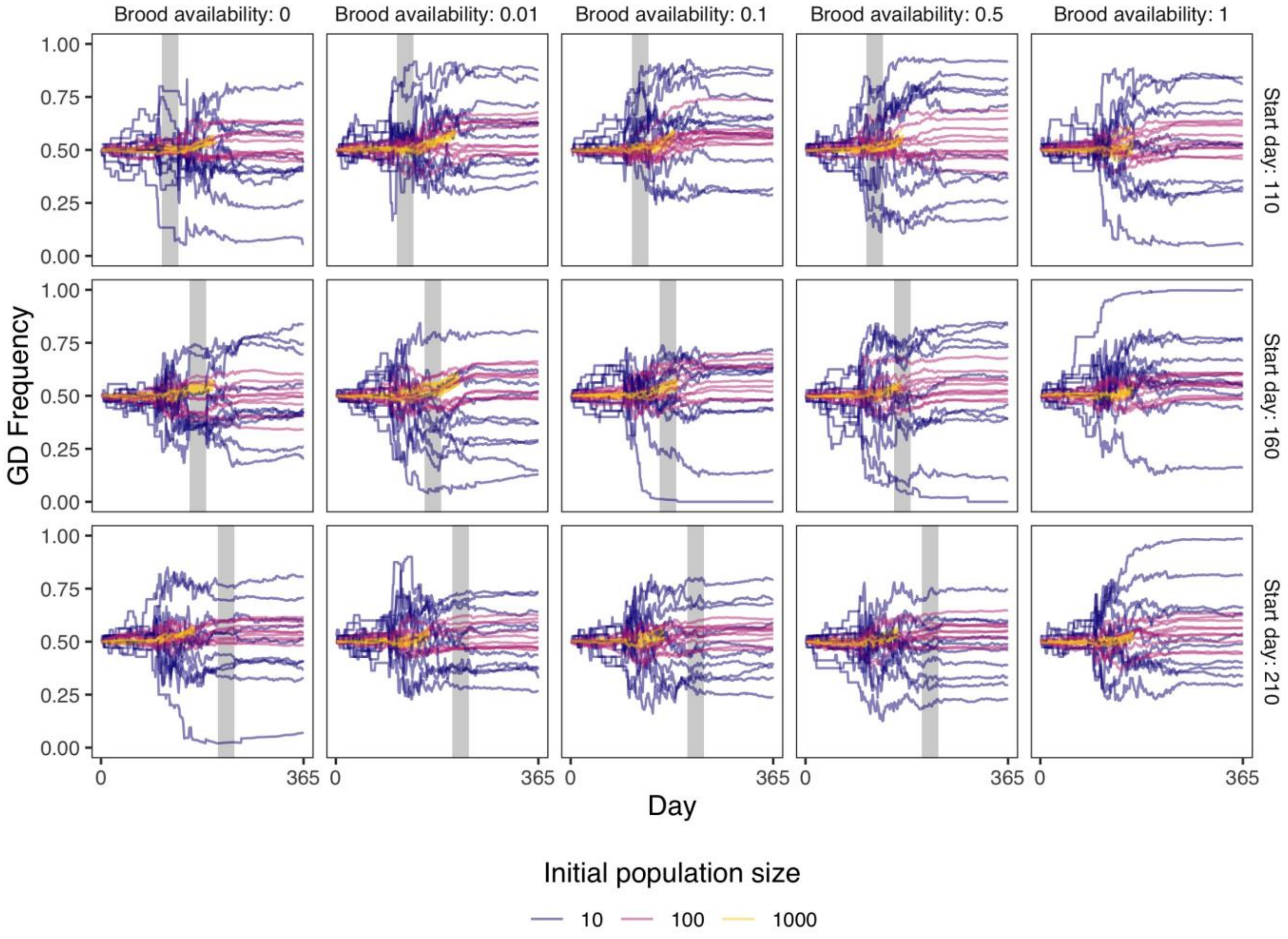

Figure S7. Gene drive (GD) allele frequency over time for different initial population sizes, given different amounts of brood cell availability (as a fraction of the normal amount) and dif f erent brood break starting days like in Figure 4. The grey bars indicate the brood break. The initial population sizes were 10, 100, or 1000 wild-type varroa with the same number of gene drive varroa on top of that, giving an initial gene drive frequency of 0.5. For every set of parameters, we run 10 repetitions and stop the model when the v arroa population size is ov er 10,000.

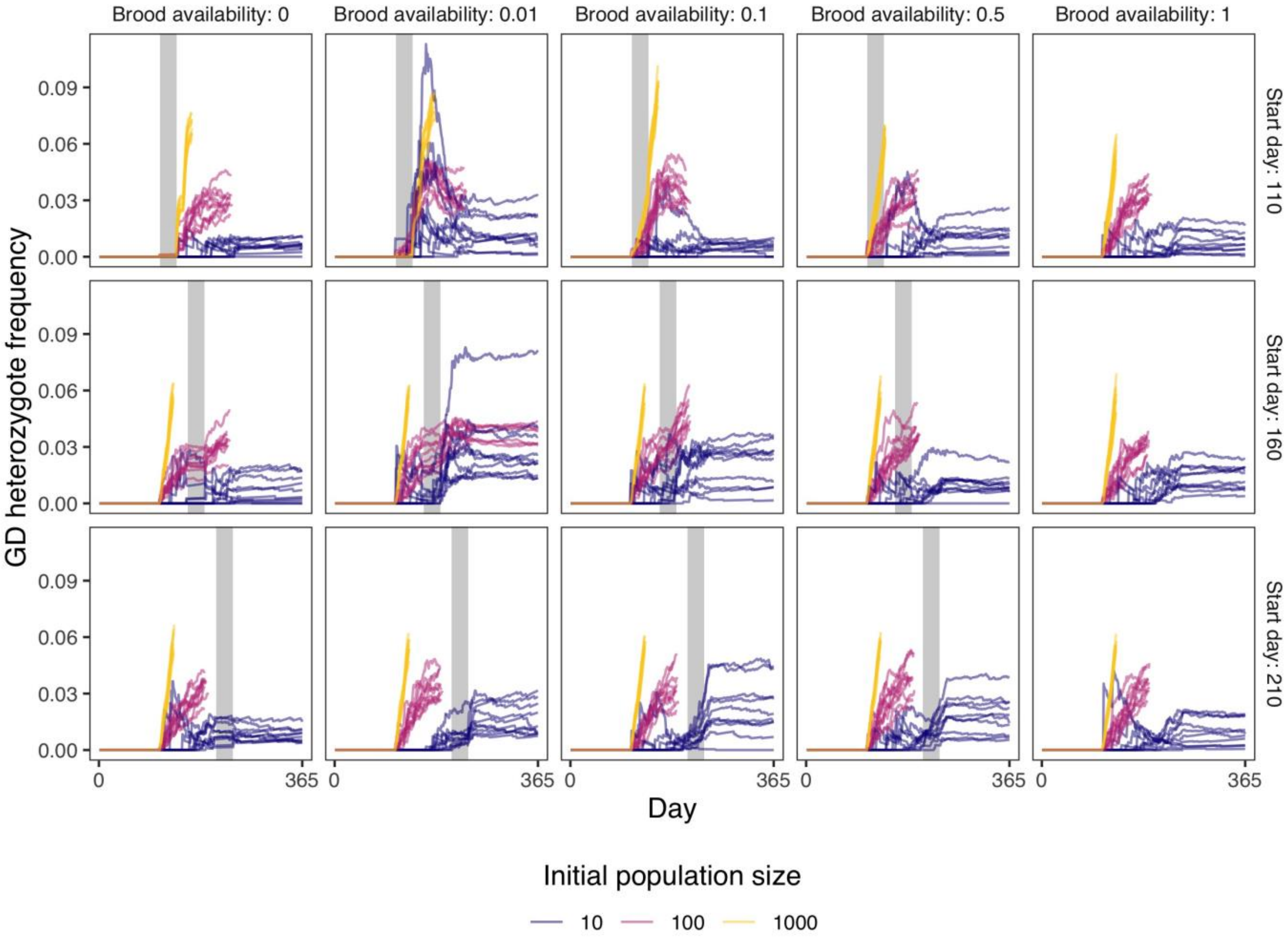

Figure S8. Gene drive (GD) heterozygote frequency over time for different initial population sizes, given different amounts of brood cell availability (as a fraction of the normal amount) and different brood break starting days like in Figure 4, but with more introduced gene drive varroa. The initial population sizes were 10, 100, and 1000 wild-type varroa with 100, 1000, and 5000 gene drive varroa on top of that, respectively, giving initial gene drive frequencies of 0.91, 0.91, and 0.83. The grey bars indicate the brood break. For every set of parameters, we run 10 repetitions and stop the model when the varroa population size is over 10,000.

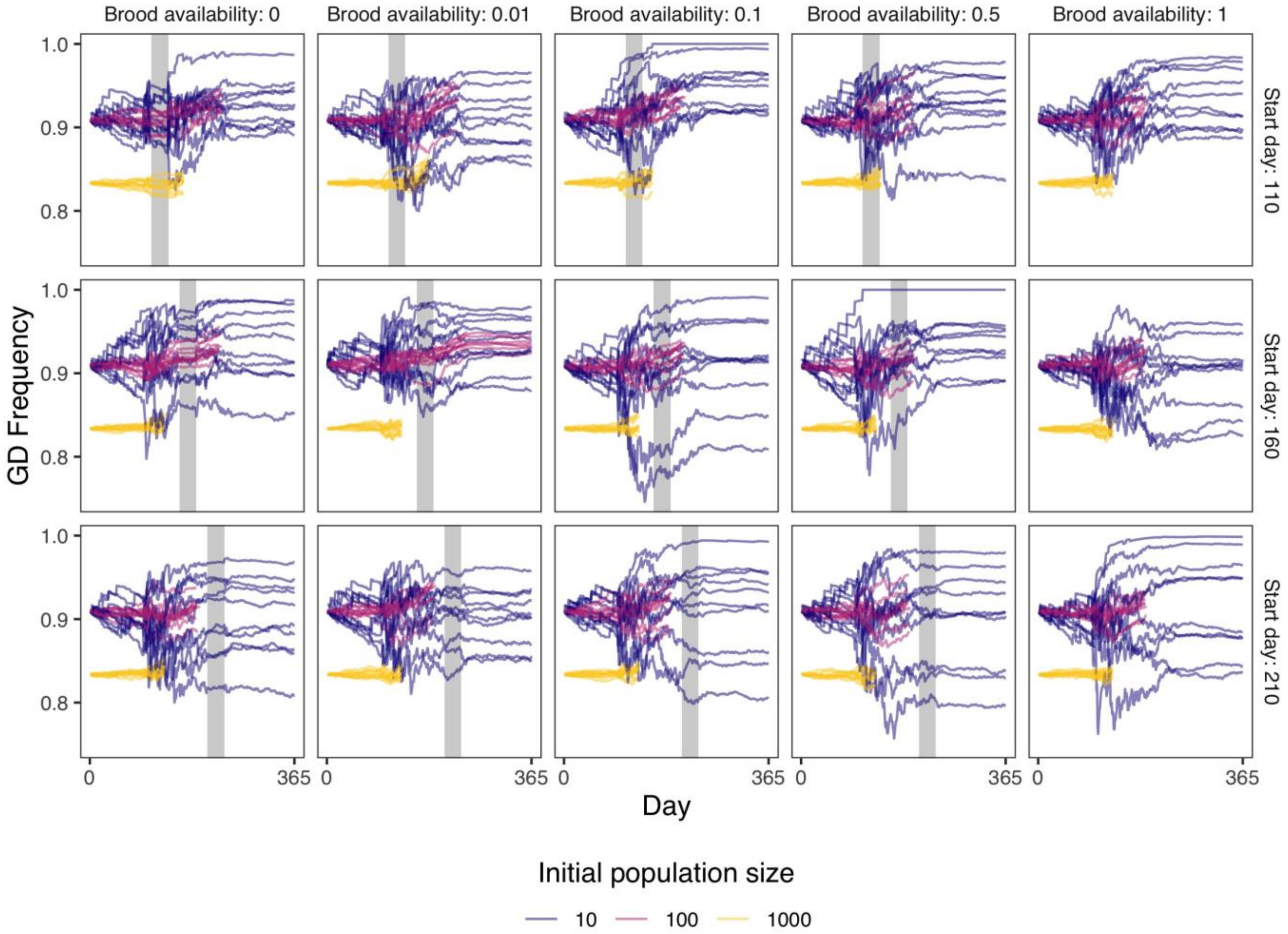

Figure S9. Gene drive (GD) allele frequency over time for different initial population sizes, given different amounts of brood cell availability (as a fraction of the normal amount) and different brood break starting days like in Figure 4, but with more introduced gene drive varroa. The initial population sizes were 10, 100, and 1000 wild-type varroa with 100, 1000, and 5000 gene drive varroa on top of that, respectively, giving initial gene drive frequencies of 0.91, 0.91, and 0.83. The grey bars indicate the brood break. For every set of parameters, we run 10 repetitions and stop the model when the varroa population size is over 10,000.

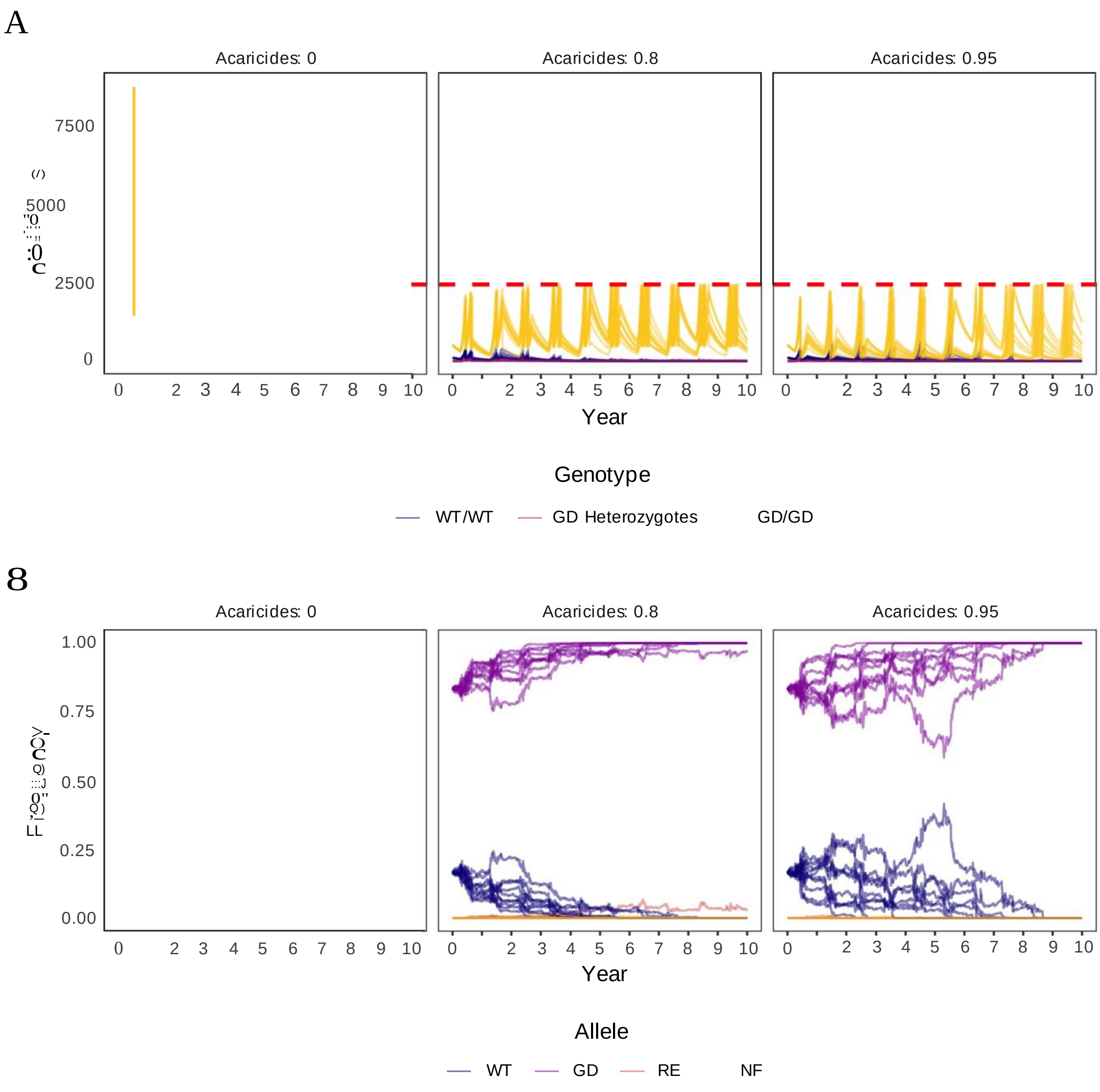

Figure 510. The spread of a gene drive while the varroa population is suppressed with acaricides whenever the varroa prevalence surpasses the danger threshold of 5% in summer (5 varroa per 100 adult bees). The same as Figure 5, but with a 10 times larger starting population. The initial population size was 100 wild-type varroa with 500 homozygous gene drive varroa, giving an initial gene drive frequency of 0.83. For every set of parameters, we run 10 repetitions and stop the model when the varroa population size is over 10,000. A) Frequencies of gene drive genotypes over time, given different intensities of acaricide treatment when the population surpasses the danger threshold. WT =wild-type, GD =gene drive. B) Frequencies of gene drive alleles over time, given different intensities of acaricide treatment when the population surpasses the danger threshold. WT =wild-type, GD =gene drive, RE = resistant, and NF = non-functional.

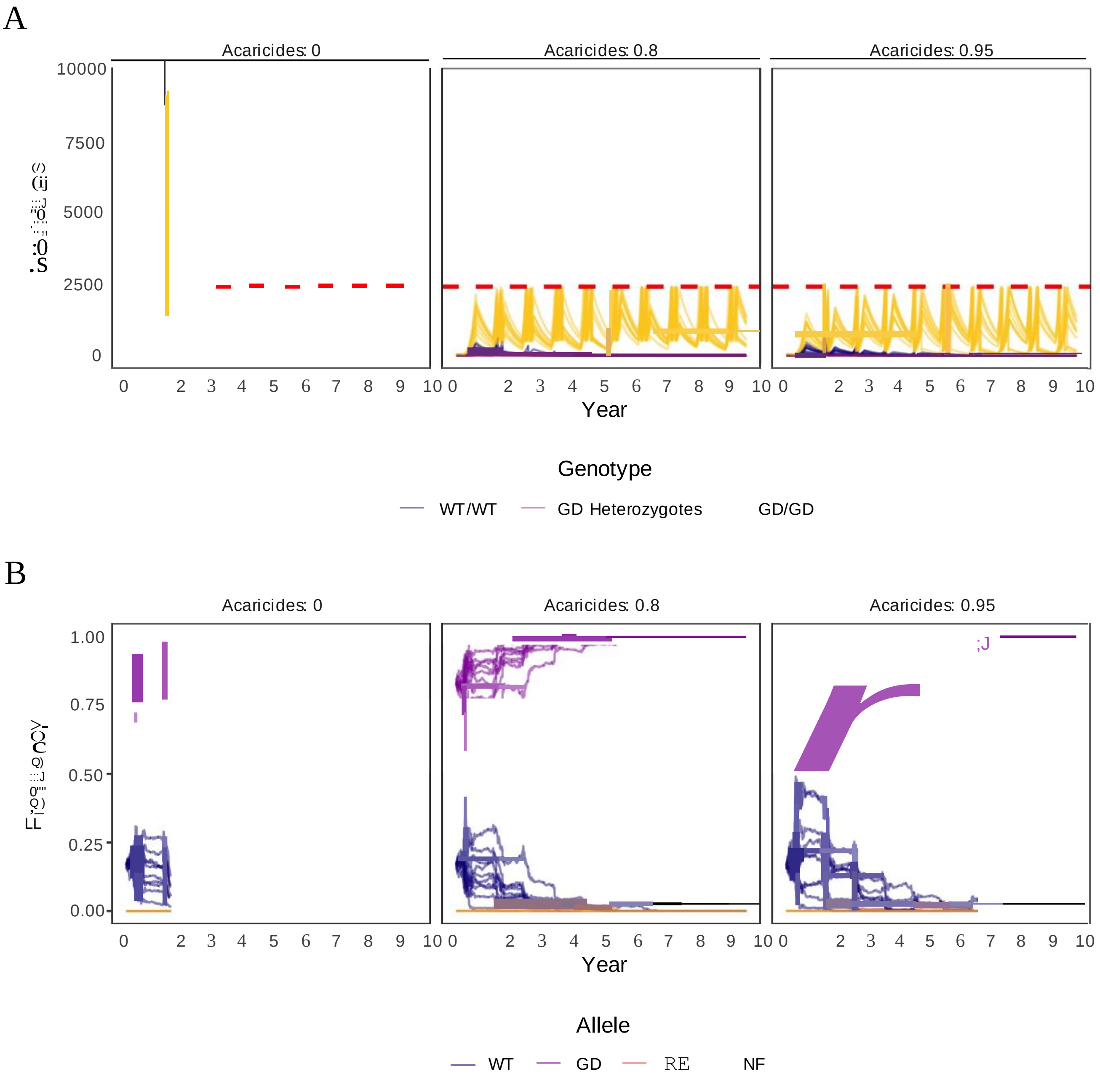

Figure 511. The spread of a gene drive while the varroa population is suppressed with acaricides whenever the varroa prevalence surpasses the danger threshold of 5% in summer (5 varroa per 100 adult bees). The same as Figure 5, but now we do an extra release of 50 gene drive varroa after every acaricide treatment. The initial population size was 10 wild-type varroa with 50 homozygous gene drive varroa, giving an initial gene drive frequency of 0.83. For every set of parameters, we run 10 repetitions and stop the model when the varroa population size is over 10,000. A) Frequencies of gene drive genotypes over time, given different intensities of acaricide treatment when the population surpasses the danger threshold. WT =wild-type, GD =gene drive. B) Frequencies of gene drive alleles over time, given different intensities of acaricide treatment when the population surpasses the danger threshold. WT =wild-type, GD =gene drive, RE =resistant, and NF =non-functiona
